# Carotid wave analysis in young adults with a history of adolescent anorexia nervosa: a case control study

**DOI:** 10.1186/s40337-023-00963-0

**Published:** 2024-02-02

**Authors:** Gabriella A. C. Springall, Greta Goldsmith, Diana Zannino, Jeanie Cheong, Jonathan P. Mynard, Michele Yeo, Michael M. H. Cheung

**Affiliations:** 1 Department of Paediatrics, University of Melbourne, Parkville, VIC Australia; 2 Heart Research, Murdoch Children’s Research Institute, Parkville, VIC Australia; 3 Clinical Epidemiology and Biostatistics Unit, Murdoch Children’s Research Institute, Parkville, VIC Australia; 4 Department of Obstetrics and Gynaecology, University of Melbourne, Parkville, VIC Australia; 5 Neonatal Services, Royal Women’s Hospital, Parkville, VIC Australia; 6 Department of Biomedical Engineering, University of Melbourne, Parkville, VIC Australia; 7 Department of Adolescent Medicine, Royal Children’s Hospital, Parkville, VIC Australia; 8 Department of Cardiology, Royal Children’s Hospital, Parkville, VIC Australia

**Keywords:** Anorexia nervosa, Wave analysis, Eating disorders, Cardiovascular risk

## Abstract

**Background:**

Anorexia nervosa (AN) is associated with abnormalities that may increase the risk of future cardiovascular disease. This study assessed the cardiovascular health of individuals who recovered from AN during adolescence by conducting wave power analysis.

**Methods:**

Former AN patients discharged from the Royal Children’s and Monash Children’s Hospitals (N = 17) in Melbourne, Australia underwent ultrasound imaging of the right carotid artery. Wave power analysis was conducted to assess biomechanical interactions of the cardiovascular system. Patient measures were compared to healthy controls (N = 51).

**Results:**

Eighty-eight percent of the former AN patients and controls were female, aged approximately 25 years, with a healthy body mass index. Mean carotid flow and pulsatility index were not different between groups. Carotid arterial strain and distensibility were lower, and the wave speed and beta stiffness index higher in the former AN patients. Characteristic impedance was not different nor were the forward and backward wave amplitudes. However, wave reflection indices (ratios of backward-to-forward compression wave area, and wave-related effect on pressure and hydraulic power) were 12–18% lower in the former AN patients (*p* < 0.05).

**Conclusions:**

Increased carotid artery stiffness and reduced wave reflection are evident in young adults who recovered from adolescent AN. This may relate to an adaptive process that helps to maintain or restore flow and characteristic impedance despite increased vessel stiffness, with this warranting future investigation.

## Abstract (plain language summary)

Anorexia nervosa (AN) is an eating disorder which may cause permanent changes in the heart and blood vessels. Blood flow properties can provide information on the health of a patient’s heart and blood vessels. In this study of young adults who recovered from adolescent AN, blood flow analysis revealed altered properties compared to controls who had never experienced an eating disorder. These alterations may help to maintain or restore blood flow despite unhealthy changes in the blood vessels themselves. Further investigation is needed to better understand how the heart and blood vessels change during and after AN to guide treatments and ongoing care. Regular assessment of the heart and blood vessels after AN recovery could identify and monitor possible health risks early.

## Background

Anorexia nervosa (AN) is associated with cardiovascular complications including decreased cardiac output, electrophysiological abnormalities, and altered blood vessel properties [1–4]. AN is the third most prevalent chronic disease in adolescent girls and is the leading cause of mortality among psychiatric disorders [5, 6], with up to 30% of deaths attributed to cardiovascular complications [7].

Existing research suggests cardiovascular risk may extend beyond a period of malnutrition and subsequent recovery, persisting into adulthood and later life. There are reports of early onset of cardiovascular disease and an increased incidence of heart disease among other populations who survived a period of malnutrition during famine or war [8–11]. Yet, the cardiovascular health of AN patients is not typically assessed following discharge from clinical services and their long-term prognosis is unknown.

Arterial wave reflection is a key contributor to ventricular afterload and has been shown to predict cardiovascular mortality [12, 13]. Wave analysis can therefore provide valuable insight into the biomechanical interactions between the heart and blood vessels. Wave intensity or wave power analysis [14, 15] is one of the most well established and widely used wave analysis techniques for investigating forward and backward waves. This technique can reveal information on cardiac function and vascular interaction by studying haemodynamic signals from a single vascular site, such as the carotid artery. The indices derived from wave analysis have been shown to provide both additional physiological insights and risk information beyond that of traditional risk factors [16–23].

Wave intensity analysis has not been performed in the AN population, yet cardiovascular complications are a major concern. We hypothesised that individuals with a history of AN would demonstrate abnormalities in carotid wave patterns compared to healthy control subjects given the increased cardiovascular risk that has been identified in malnourished populations. The aim of this study was to extend our previous work investigating the long-term cardiovascular risk following AN recovery [24], by assessing biomechanical cardiovascular interactions in former patients using wave analysis techniques. This can assist early identification and treatment of cardiovascular abnormalities.

## Methods

### Study design and sample

Using a cross-sectional study approach, we recruited a group of young adults (N = 17) who were treated for adolescent AN (Diagnostic and Statistical Manual of Mental Disorders classification) [25] and clinically discharged from the Royal Children’s Hospital or Monash Children’s Hospital Eating Disorder Service, Melbourne between June 2008 and July 2016. Patients were eligible for inclusion if they had been discharged from the Eating Disorder Service five or more years ago, were aged 18 years and over, exhibited an Eating Disorder Examination Global Score within 1SD of community norms (i.e. they no longer exhibited features of an eating disorder), and possessed a body mass index (BMI) ≥ 18.5 kg/m^2^. Healthy age- and sex-matched controls (N = 51) were drawn from the Victorian Infant Collaborative Study (VICS) [26] control cohort, with a ratio of 3:1.

A sample size of 68 participants (17 former AN and 51 control) would enable differences of at least 0.7SD to be detected with 80% power using a two-sided type I error of 5%.

### Study procedures

Participants laid supine with the head turned 45° to the left while an ultrasound of the right carotid artery was performed with a GE Vivid ultrasound machine (Vivid i BT06, 10–15 MHz linear array probe; GE Healthcare). Arterial diameter, pressure, and flow velocity waveforms were obtained from Doppler and M-mode images.

A diameter waveform ($D$) was obtained via semi-automated segmentation of the upper and lower vessel walls from the M-mode images. A pressure waveform ($P$) was then obtained by calibrating the diameter waveform to central mean and diastolic pressures [27]. Central blood pressure was estimated from peripheral measurement of the brachial blood pressure in the non-dominant arm using in-built functions in the SphygmoCor XCEL system (Atcor Medical, Sydney, Australia). Velocity waveforms were obtained by semi-automated segmentation of the upper and lower envelopes of the Doppler spectrum, with mean velocity (obtained as the intensity-weighted spectral mean) used for analysis [27]. After applying a velocity correction factor, as described by Kowalski and colleagues [27], volumetric blood flow waveforms ($Q$) were calculated as the product of velocity (${U}_{mean}$) and arterial cross-sectional area ($A= \pi {D}^{2}/4$):$Q= {U}_{mean}A$

Carotid artery flow is a vital contributor to cerebral haemodynamics due to the carotid arteries carrying over 80% of the cerebral blood supply [28].

Pulsatility index was calculated from the ratio of velocities, with this being a measure of vascular resistance distal to the carotid artery: $Pulsatility \space Index= \frac{{U}_{max}-{U}_{min}}{{U}_{mean}}$  

Measures of wall mechanics were also calculated from diameter, area, and pressure parameters as follows.

$$Strain = \frac{{D_{max} - D_{min} }}{{D_{min} }}$$

$$Distensibility = \frac{{A_{max} - A_{min} }}{{A_{min} \left( {P_{max} - P_{min} } \right)}}$$

$${\text{Bramwell}} - {\text{Hill}}\;{\text{Wave}}\;{\text{Speed}},\;C_{BH} = \sqrt {\frac{{A_{min} }}{\rho }\frac{SBP - DBP}{{A_{max} - A_{min} }}}$$

$${\text{Beta}}\;{\text{Stiffness}}\;{\text{Index}},\;\beta = \frac{{{\text{ln}}\left( {SBP/DBP} \right)}}{strain}$$

where $\rho$ is blood density (1.06 g/cm^3^).

Characteristic impedance (${Z}_{c}$) was calculated via the slope of the early $P$-$Q$ relation [29, 30]. These measures indicate arterial stiffness and thereby the degree of vascular aging [9].

Time-corrected wave power (*wp*) was calculated from pressure and flow as *wp* = (*d* $$P/dt)($$ *d* $$Q/dt)$$ [15]; this quantity is similar to wave intensity, which uses velocity rather than flow, but unlike wave intensity is conserved at junctions. Forward ( +) and backward (–) components of wave power were calculated as $${\text{w}}{p}_{\pm }=\pm \left(dP/dt\pm {Z}_{c}dQ/dt\right)/\left(4{Z}_{c}\right)$$. Pressure and flow were likewise separated into components via $${P}_{\pm }=\left(P\pm {Z}_{c}Q\right)/2$$ and $${{\text{Q}}}_{\pm }=\left(Q\pm P/{Z}_{c}\right)/2$$. Finally, hydraulic pressure power was calculated as $$\Pi =PQ$$ and was also separated into forward and backward components via $${\Pi }_{\pm }={P}_{\pm }{Q}_{\pm }$$ [15].

The carotid wave intensity signal generally exhibits four main waves [31]: an initial pressure- and flow-increasing forward compression wave (FCW) arising from blood acceleration following aortic valve opening; a pressure-increasing flow-decelerating backward compression wave (BCW) arising from wave reflection in the distal vascular bed; a late systolic pressure- and flow-decreasing forward decompression wave (FDW) associated with flow deceleration prior to valve closure, and a mid-systolic forward decompression wave (FDWx) thought to arise from negative re-reflection of the BCW when this backward-running wave encounters the brachiocephalic trunk and/or aorta. The amplitude of each wave was quantified via its peak wave power (PWP), area, pressure effect (Δ $P$), and hydraulic power effect (ΔΠ). Wave reflection was quantified via the BCW-FCW area, Δ $P$, and ΔΠ ratios [31].

### Statistical analysis

General descriptive statistics of the former AN patients and controls were calculated. Body surface area was calculated using the DuBois formula [32]. Clinical data relating to the severity and duration of malnutrition obtained at the time of diagnosis were also summarised for the former AN patients. Mean carotid flow was indexed for height. Mean and standard deviation (SD) were reported for normally distributed data, or the median and interquartile range (IQR) for non-normally distributed data. Comparisons were made between former patients and controls using the Wilcoxon test with group as the variable of interest. For each analysis output, *p*-values less than or equal to 0.05 were considered statistically significant. All statistical analyses were performed using R software (version 4.0.1, R Foundation, Vienna, Austria).

## Results

### Population

Descriptive statistics of the former AN patients (N = 17) and healthy controls (N = 51) are presented in Table 1. The former AN patients and controls had an average age of 25 years and similar BMI and body surface areas. The BMI of both groups was within the healthy range indicating healthy body fatness [28]. Most (88%) of the former AN patients were female, all of whom had regular menstrual periods. The time interval since discharge from their respective eating disorder services ranged from 5–10 years with a mean (SD) interval of 7.4 (2.0) years.

**Table 1:** Characteristics of the study sample

	Former AN (N = 17)	Healthy Controls (N = 51)
Age, years	24.7 (2.5)	25.0 (0.8)
Female	15 (88%)	45 (88%)
Height, cm	169.1 (7.4)	170.1 (5.6)
Weight, kg	62.0 (9.4)	67.9 (6.7)
BMI, kg/m^2^	21.68 (1.90)	23.47 (1.52)
BSA, m^2^	1.71 (0.16)	1.79 (0.17)

Data are N (%) (categorical) or mean (SD) (continuous)

At the time of diagnosis the former AN patients had a mean (SD) age of 15.8 (1.7) years and BMI of 16.1 (1.9) kg/m^2^. This indicates a moderately malnourished cohort [25]. The patients had lost an average of 25% (ranging from 10–37%) of their body weight over 5–24 months.

### Wave power analysis

There was a trend towards higher mean carotid flow in the former AN patients (*p* = 0.071) that was also present when indexed for height (*p* = 0.083, Table 2), but the magnitude of the difference for indexed carotid flow was small (2%). There was some evidence of a small (~ 10%) difference in pulsatility index (*p* = 0.081). There was stronger evidence of a difference in carotid arterial stiffness (indicative of vascular aging), with the former AN patients exhibiting lower strain (*p* = 0.028), lower distensibility (*p* = 0.067), higher Bramwell-Hill wave speed (*p* = 0.061), and a higher Beta stiffness index (*p* = 0.038). There was no evidence of a difference in the characteristic impedance (${Z}_{c}$) between groups (*p* = 0.817).

**Table 2:** Carotid flow and wall mechanics of former patients and controls

	Former AN (N = 17)	Healthy Controls (N = 51)
Carotid flow		
Mean flow, mL/min	504 (456, 528)	450 (348, 528)
Mean flow, mL/min/m	276 (264, 324)	270 (210, 318)
Pulsatility Index, Mean (SD)	2.0 (0.4)	2.2 (0.4)
Wall mechanics		
Strain, %	13.8 (12.8, 15.8)*	16.3 (14.4,18.1)
Distensibility, 1/mmHg	0.012 (0.011, 0.014)	0.014 (0.011, 0.017)
Bramwell–Hill Wave Speed, cm/s	324.7 (294.6, 343.1)	296.7 (270.6, 331.6)
Beta Stiffness Index	2.2 (2, 2.3)*	1.9 (1.7, 2.3)
Z_c_, g/s/cm^4^, Mean (SD)	1412.3 (302.6)	1391.4 (364.4)

Data are median (IQR), unless specified otherwise; *p < 0.05

**Fig. 1:**
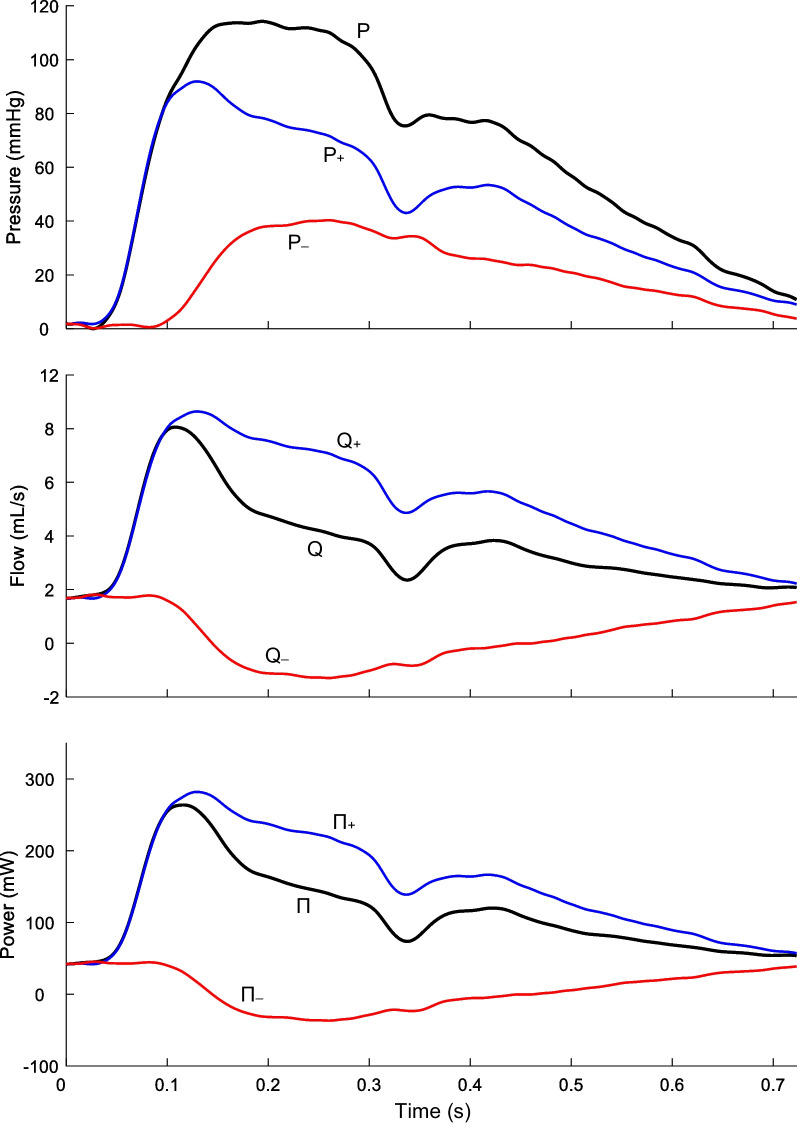
Former AN patient carotid pressure (P), flow (Q), and hydraulic power (Π) waveforms. Forward components (P_+_, Q_+_, and Π_+_) and backward components (P_−_, Q_−_, and Π_−_)

**Fig. 2:**
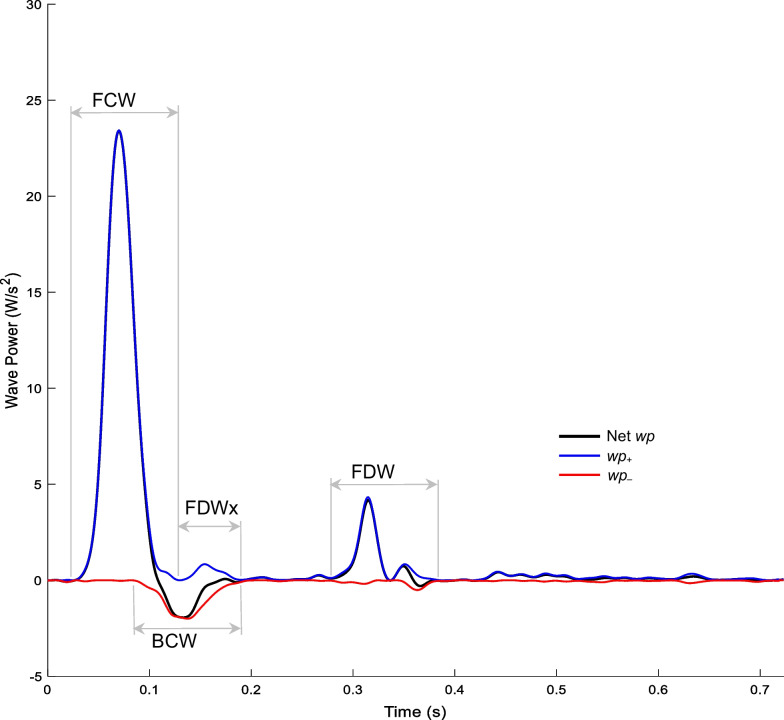
Former AN patient wave power analysis forward compression wave (FCW), backward compression wave (BCW), and forward decompression wave (FDW). Forward and backward wave power (*wp*) components (*wp*_+_ and *wp*_−_)

There was no evidence of a difference in the amplitudes of FCW, BCW, FDW or FDWx between former AN patients and controls (Table 3). However, former AN patients exhibited 12–18% lower BCW-FCW area, pressure, and power ratios (*p* = 0.032, 0.029, and 0.009, respectively). Figure 1 provides a representative example of components of the pressure, flow, and hydraulic power waveforms of a former AN patient; while Fig. 2 shows an example of the wave power analysis.

**Table 3:** Carotid PQ wave analysis of former patients and controls

	Former AN (N = 17)	Healthy Controls (N = 51)
FCW		
PWP, W/s^2^	20.5 (16, 31.8)	19.8 (13.3, 29.4)
Area, W/s	0.7 (0.5, 1)	0.7 (0.5, 1)
ΔP_fw_, mmHg, Mean (SD)	20.2 (4.4)	18.7 (4.6)
ΔΠ_fw_, mW	227.1 (182.3, 291)	217.3 (162.4, 275.7)
BCW		
PWP, W/s^2^	− 4.2 (− 5.2, − 1.9)	− 3.5 (− 5.8, − 2.5)
Area, W/s	− 0.1 (− 0.2, − 0.1)	− 0.1 (− 0.2, − 0.1)
ΔP_bk_, mmHg	9.3 (7.6, 10.7)	8.8 (7.9, 10.5)
ΔΠ_bk_, mW	− 86.9 (− 105.4, − 65.8)	− 84.8 (− 102.9, − 72)
FDW		
PWP, W/s^2^	2.7 (2, 3.1)	2.2 (1.4, 2.8)
Area, W/s	0.1 (0, 0.1)	0.1 (0, 0.1)
ΔP_fw_, mmHg	− 6 (− 7.9, − 5.5)	− 5.6 (− 7.5, − 4.8)
ΔΠ_fw_, mW	− 89.5 (− 97.8, − 63.8)	− 67.3 (− 89.4, − 55.8)
FDWx		
PWP, W/s^2^	1.2 (0.9, 2.8)	1.8 (1, 3.4)
Area, W/s	0.1 (0, 0.1)	0 (0, 0.1)
ΔP_bk_, mmHg	− 4.1 (− 5.8, − 3.7)	− 4.3 (− 5.8, − 3.3)
ΔΠ_bk_, mW	− 52.3 (− 76.1, − 45.9)	− 59.6 (− 96.7, − 36.5)
Wave Ratios		
BCW Area/FCW Area	− 0.163 (− 0.189, − 0.136)*	− 0.189 (− 0.270, − 0.160)
BCW ΔP_bk_/FCW ΔP_fw_, Mean (SD)	0.453 (0.079)*	0.517 (0.129)
BCW ΔΠ_bk_/FCW ΔΠ_fw_, Mean (SD)	− 0.352 (0.071)*	− 0.427 (0.132)

Data are median (IQR), unless specified otherwise; *p < 0.05

FCW, forward compression wave; BWC, backward compression wave; FDW, forward decompression wave; PWP, peak wave power; ΔP_fw_ and ΔP_bk_ are the wave-related changes in forward and backward components of pressure respectively; ΔΠ_fw_ and ΔΠ_bk_ are the wave-related changes in forward and backward components of hydraulic power respectively

## Discussion

In this first study of carotid wave analysis of former AN patients we have observed comparable blood flow and characteristic impedance, but higher carotid arterial stiffness. Forward and backward wave amplitudes were no different, but lower wave ratios suggested lesser wave reflection. The differences support our hypothesis that individuals with a history of AN would exhibit abnormalities in wave patterns compared to healthy controls.

Our data suggests that any reduction in mean cerebral flow (assessed by carotid flow in this study) during malnutrition was not sustained long-term in the former AN patients. This differs from a prior study that reported persistently reduced regional cerebral blood flow four years post treatment for adolescent AN [33]. That study measured cerebral flow via single positron emission computed tomography, whereas we measured carotid flow via ultrasound. Furthermore, our assessment time point was an additional 1–6 years beyond recovery; allowing potentially more time for cardiovascular normalisation. Further work would be needed to establish whether the differing findings arise from differences in methodology, patient characteristics, length of follow-up or a combination of these.

Lower BCW-FCW area, pressure, and power ratios signify lower cerebral wave reflection in the former AN patients. Bleasdale and colleagues suggested that less wave reflection may indicate reduced cerebral vasomotor tone [34]. A decrease in cerebral tone was identified during acute hypercapnia in a single population, and therefore it is unclear whether differences in baseline wave reflection in two groups may be accounted for by such differences in cerebral tone. Our data could suggest that differences in baseline tone exist between former AN patients and controls rather than arise from the period of malnutrition. In addition, less wave reflection promotes flow and could have contributed to the maintenance or normalisation of cerebral flow in former AN patients over time.

Reduced cerebral wave reflection has also been observed in healthy adults in response to acute inflammation [35]. Although there is evidence of elevated inflammatory markers in AN patients at the time of malnourishment [36], these typically normalise throughout recovery and the possible cardiovascular implications post recovery are unknown. Schroeder and colleagues suggested that lower wave reflection may increase the brain’s vulnerability to pulsatile haemodynamics [35]. Another study found lower carotid reflection index, pulsatility index, and mean velocity in older versus younger adults [37]. Our data may therefore imply that the former AN patients have a more ‘aged’ carotid/cerebral arterial network (consistent with increased carotid stiffness). However, our group has recently shown that although wave reflection promotes transmission of pulsatile pressure, it impedes pulsatile power transmission, to distal vascular beds [38]. Whether the lower wave reflection seen in former AN patients is potentially beneficial or detrimental is therefore unclear.

Our data revealed no difference in the FCW or FDW amplitude, which suggest systolic and diastolic function of the left ventricle are normal in the former AN patients [39]. Previous studies, using standard echocardiography and tissue Doppler imaging, have reported no diastolic abnormalities in AN patients at the time of malnourishment [40]; with this being supportive of our wave intensity findings. Furthermore, higher FCW intensity has been associated with cognitive decline in mid- to late-life [23]. Our observation of no difference in FCW between groups therefore implies recovered AN haemodynamics may not confer greater risk of cognitive decline in later life.

Lastly, measures of wall mechanics are consistent with previous findings in this population [24] in which the former AN patients had stiffer carotid vessels. However, the lack of change in characteristic impedance (${Z}_{c}$) indicates that the pressure-flow relationship is maintained despite increased carotid stiffness. Whilst this could be explained by an increase in the vessel size [15], we previously found no differences in the diameter of the carotid artery between former AN patients and controls [24], and therefore more detailed investigation of this finding is needed in future studies.

As our findings are specific to young adults who received specialist treatment and recovered from AN during adolescence, these may have limited generalizability to other populations. In addition, clinical data collected at the time of diagnosis indicate our AN population had not experienced the greatest degree of illness severity or chronicity. Our results may therefore reflect more favourable long-term outcomes than patients who had been severely malnourished or the period of malnutrition extended beyond two years. Furthermore, this study was not powered to detect sex differences in wave intensity and males and females may be affected differently. Further investigation in other populations who experienced malnutrition at different stages of growth or development and for varying durations, or recurrent episodic periods of malnutrition, would provide valuable insights into long-term cardiovascular risk from AN. Independent assessment of male and female cohorts may also be useful to identify sex-dependent cardiovascular risk, especially given the number of males afflicted is increasing [41].

This study draws attention to ongoing cardiovascular abnormalities in former AN patients. Regular assessment of blood pressure to test both the hearts pumping capacity and arterial stiffness, preferably alongside direct assessment of atrial stiffness via tonometry or ultrasound, could assist early identification of premature vascular aging or cardiovascular disease. This could allow intervention to optimise the duration and quality of a patients life. Ultimately, future research aimed at understanding the mechanisms underlying altered haemodynamics in AN and monitoring cardiovascular normalisation and maintenance (using both standard measures of blood pressure and heart rate as well as assessing arterial stiffness) will be pivotal to optimising treatment regimes and patient outcomes.

## Conclusions

In conclusion, although ventricular function does not appear to be affected in the long-term in individuals with a history of adolescent AN, accelerated carotid vascular aging and altered cerebral vasomotor tone exist. When combined with our previous findings of altered large artery stiffness and abnormalities in endothelial function and autonomic control [24], the evidence suggests a period of significant malnutrition during adolescence due to AN has cardiovascular consequences which last into adulthood. Former AN patients may experience adaptive processes, involving reduced wave reflection, that help to maintain cerebral flow. This draws attention to the need for regular assessment of cardiovascular health in former AN patients.

## Data Availability

The datasets used and analysed during the current study are available from the corresponding author upon reasonable request.

## Abbreviations

AN: Anorexia nervosa
BCW: Backward compression wave
BMI: Body mass index
FCW: Forward compression wave
FDW: Forward decompression wave
FDWx: Mid-systolic forward decompression wave
PWP: Peak wave power
VICS: Victorian Infant Collaborative Study
